# Generalized linear mixed-effects models for studies using different sets of stimuli across conditions

**DOI:** 10.3389/fpsyg.2022.955722

**Published:** 2022-09-02

**Authors:** ShunCheng He, Wooyeol Lee

**Affiliations:** Department of Psychology, Chungbuk National University, Cheongju, South Korea

**Keywords:** generalized linear mixed-effects model (GLMM), experimental data analysis, non-repeated item (NRI) design, Monte Carlo simulation, Type I error, power

## Abstract

A non-repeated item (NRI) design refers to an experimental design in which items used in one level of experimental conditions are not repeatedly used at other levels. Recent literature has suggested the use of generalized linear mixed-effects models (GLMMs) for experimental data analysis, but the existing specification of GLMMs does not account for all possible dependencies among the outcomes in NRI designs. Therefore, the current study proposed a GLMM with a level-specific item random effect for NRI designs. The hypothesis testing performance of the newly proposed model was evaluated via a simulation study to detect the experimental condition effect. The model with a level-specific item random effect performed better than the existing model in terms of power when the variance of the item effect was heterogeneous. Based on these results, we suggest that experimental researchers using NRI designs consider setting a level-specific item random effect in the model.

## Introduction

Generalized linear mixed-effects models (GLMMs; Stroup, 2012) have been widely applied in various contexts in psychology (Judd et al., 2012; Moscatelli et al., 2012; Trippas et al., 2017). The use of GLMMs for experimental data analysis was proposed a decade ago (Baayen et al., 2008; Barr, 2008; Quené and Van den Bergh, 2008) and is now widely accepted (Goldhammer et al., 2017; Cho et al., 2018; Singmann and Kellen, 2019). GLMMs are useful for experimental data because they can include all sources affecting the responses in a model with fixed or random effects, and the distribution of responses is not limited to a normal distribution. For example, this model can include the effects of the experimental condition as a fixed effect while the variability of participants or items as a random effect. Additionally, binary dependent variables are explained as the sum of the effects on the logit scale.

In within-participant experimental designs, the dependent variable is measured in a group of participants at every level of an independent variable of interest. This type of design can be considered a hierarchical data structure where the repeatedly measured responses are nested within participants. In psychological experiments, however, the dependent variable is often measured using several items from each participant in each level of experimental condition. In this case, the data structure is no longer hierarchical, but the responses are *cross-classified* by the participants and items.

In a cross-classified design, items can also be included as a component of the experimental design. For example, between-items design refers to an experimental design in which items are not repeatedly presented between levels of an experimental condition (Barr et al., 2013). This design can be used to avoid learning effects or mere-exposure effects (Gordon and Holyoak, 1983). The present study defines the experimental design combining within-participant design and between-items design as a non-repeated item (NRI) design. Table 1A depicts an example dataset of an NRI design in long format. In this example, the participants are exposed to the bug and fruit levels of an experimental condition (i.e., category). At each level, the dependent variable is measured using multiple items. The items used at the two levels are samples from different populations, so items are not repeated over the levels.

**TABLE 1:** Example dataset (A) and its mean response (B) for non-repeated item (NRI) design.

(A)			
**Participant ID**	**Item**	**Category**	**Response**
P1	Ants	Bug	0
P1	Cricket	Bug	1
P1	Bees	Bug	0
P1	Grape	Fruit	1
P1	Melon	Fruit	1
P1	Apple	Fruit	0
P2	Ants	Bug	0
P2	Cricket	Bug	0
P2	Bees	Bug	0
P2	Grape	Fruit	1
P2	Melon	Fruit	1
P2	Apple	Fruit	0
P3	Ants	Bug	0
P3	Cricket	Bug	1
P3	Bees	Bug	0
P3	Grape	Fruit	1
P3	Melon	Fruit	1
P3	Apple	Fruit	1

**(B)**			

**Participant ID**	**Category**		**Mean response**

P1	Bug		0.33
P1	Fruit		0.67
P2	Bug		0.00
P2	Fruit		0.67
P3	Bug		0.33
P3	Fruit		1.00

NRI design is commonly used in psychological research. A literature survey was conducted to provide evidence for the claim. All papers published in 2021 were reviewed in the 150th volume of the Journal of Experimental Psychology: General, one of the APA journals for experimental psychology research. The survey results showed that 25 out of the 145 papers (17%) were based on the NRI design. Seven papers included at least one binary dependent variable out of the 25 papers where the NRI design was used. Sixteen papers used repeated measures analysis of variance (RM-ANOVA) only and one paper reported Bayes factors in addition to RM-ANOVA results. Nine papers used the GLMM framework, but no study considered the item variance heterogeneity in the model.

However, NRI designs have rarely been mentioned in the relevant literature. One reason may be the widespread practice that the RM-ANOVA has been the primary analysis method used for NRI designs. As shown in Table 1B, in the ANOVA framework, the dependent variable is redefined as the mean response across items for each participant in each level of the experimental condition, and subsequent analyses are performed with the newly defined dependent variable. This practice is based on two assumptions. First, the variability of the item effects does not differ between the levels of the experimental condition. Second, the measurement error of the mean response as an estimate becomes negligible as the number of the items increases (Luck, 2005). However, if the variance of the item effect varies for each level and is ignored, the RM-ANOVA cannot handle this item effect heterogeneity. In psycholinguistics, by-participant and by-item tests (called *F*1/*F*2 tests, respectively) have been conventionally reported (Clark, 1973; Raaijmakers et al., 1999; Raaijmakers, 2003). According to this approach, the mean difference between levels is considered significant only when both *F*1 and *F*2 tests reveal a significant result. However, this convention cannot avoid inflated Type I and Type II errors related to the effect of experimental conditions (Raaijmakers et al., 1999; Baayen et al., 2008; Barr et al., 2013).

Among current GLMMs being employed for experimental data analysis, the fullest model specifies a random intercept and a random slope for both participants and items (Barr et al., 2013; Matuschek et al., 2017). However, this model is unsuitable for NRI design datasets because, unlike the participants, the items do not overlap across the levels. On the other hand, a model in which a random intercept is specified for the random effect of all items cannot cope with the heterogeneity of item effects. As in the RM-ANOVA, if the random effect structure is not well established in the GLMM, the inference about the fixed effect will be inaccurate (Verbeke and Lesaffre, 1997; Litière et al., 2007). Therefore, it is necessary to specify a level-specific item random effect for NRI designs.

GLMMs are estimated using maximum likelihood (ML) or Bayesian approaches, which can now be easily estimated in R (Lee and Grimm, 2018). Several hypothesis testing methods are used for fixed effects when ML is chosen as an estimation method. Both likelihood ratio (LR) and Wald tests use a theoretical distribution such as the standard normal (*Z*) or chi-square (*χ*^2^) as a reference distribution, but the logic for calculating these statistics is different (Molenberghs and Verbeke, 2007). While both are assumed to follow the theoretical distribution asymptotically, the sample size of the experimental data is relatively small. Accordingly, we examined the performance of the LR and Wald tests in GLMMs for experimental data.

The primary purpose of this study is to investigate the consequences of misspecifying item random effects in NRI designs. The remainder of this paper is organized as follows. First, two GLMMs are introduced that can be used for NRI designs. Second, an illustrative example of using a GLMM for an NRI design is provided. Third, the performance of the hypothesis testing of the two GLMMs is examined through a simulation study. Finally, a discussion is presented.

## Generalized linear mixed-effects models for non-repeated item designs

In this study, NRI designs are explained using the following situation. A binary dependent variable is measured at each level *k* of an experimental condition with two levels (*K* = 2), and *J* participants are exposed to both levels. The dependent variable is measured with *I* items at each level, and the items do not overlap between the levels. Thus, the total number of items is 2 _×_ *I.* Two GLMMs in the mathematical form are presented below, and the lme4 (Bates et al., 2015) syntax corresponding to each is shown in Table 2. The first model is the common item random effect model. This model is equivalent to the participant random slope model in the previous literature (Barr et al., 2013). The effect of all items is assumed to follow a single distribution. The second model is the level-specific item random effect model, which adds complexity to the first model. This model includes the variance parameters of the item random effect for each level of the experimental conditions in the NRI design.

**TABLE 2:** *lme4* specification of GLMMs in this study.

Model	*lme4* specification
M1	y ∼ x + (1+x| participant) + (1| item)
M2	y ∼ x + (1+x| participant) + (−1+c1| item) + (−1+c2| item)

M1, Common item random effect model; M2, Level-specific item random effect model. “c1” and “c2” represent indicator variables for each level of the experimental conditions.

### Model specification

#### Common item random effect model (M1)

Let *y*_*jik*_ be the response from participant *j* (*j* = 1, …, *J*) on item *i* (*i* = 1, …, 2*I*) in the *k*th level (*k* = 1 and 2) of the experimental condition. The equation for the common item random effect model (M1) for an NRI design can be written as:


(1) $$logit\left[P\left(y_{jik}=1\right)\right]=\beta_{0}+\beta_{1}x_{k}+s_{0j}+s_{1j}x_{k}+w_{i}$$

The response *y*_*jik*_ is explained on the logit scale using the right term of the equation. *x*_*k*_ is the experimental condition with dummy coding. That is, the first and second levels are coded as 0 and 1, respectively. *β*_0_ and *β*_1_ are the fixed effects for the intercept and the slope, respectively. The fixed effect refers to the effect of the experimental condition on average participants and items. The expressions intercept and slope are taken from multilevel modeling (Snijders and Bosker, 2012). Since the experimental condition is a categorical independent variable, the intercept refers to the mean at the first level, the slope refers to the mean difference between the first and second levels of the experimental condition.

*s*_0*j*_ and *s*_1*j*_ are the participant random intercept and the participant random slope of participant *j*, respectively; and *w*_*i*_ is the item random effect of item *i*. The random effect refers to the unique effect of an individual participant or item. Since individual responses are cross-classified by both the participant and the item simultaneously, the participant random effect can be seen as the average effect of individual participant across items within the experimental condition, and the item random effect as the average effect of the items across participants.

The participant and item random effects are assumed to be distributed as in (2) and (3), respectively:


(2) $$\left[\begin{array}{c} s_{0j}\\ s_{1j} \end{array}\right]∼MVN\left(\left[\begin{array}{c} 0\\ 0 \end{array}\right],\left[\begin{array}{cc} \tau_{0}^{2} & \tau_{01}\\ \tau_{10} & \tau_{1}^{2} \end{array}\right]\right)\mathrm{and}$$

(3) $$w_{i}∼N\left(0,\omega^{2}\right)$$

#### Level-specific item random effect model (M2)

The equation for the level-specific item random effect model (M2) is:


(4) $$logit\left[P\left(y_{jik}=1\right)\right]=\beta_{0}+\beta_{1}x_{k}+s_{0j}+s_{1j}x_{k}+w_{ik}$$

The model specification for M2 is identical to that for M1, except that the item random effect _*w_ik_*_ has an additional subscript *k*, indicating it is a level-specific item random effect. This effect follows a normal distribution for each level, as shown in (5):


(5) $$w_{i1}∼N\left(0,\omega_{1}^{2}\right)\mathrm{and}w_{i2}∼N\left(0,\omega_{2}^{2}\right)$$

M1 and M2 are nested models. M1 can be considered a particular case when the variance of level-specific item random effect of M2 is equal between the levels (i.e., $\omega_{1}^{2}=\omega_{2}^{2}$). Logically, M2 is the most complex model for NRI design. One might consider the model having a random slope for item effect as a more complex model. However, in the NRI design, the random slope cannot be specified in the model, unlike the participants, because the items are not used repeatedly between the levels of the experimental condition.

### Estimation and hypothesis testing methods

In this study, the model is estimated using the *glmer* function in R’s *lme4* package (Bates et al., 2015), which uses an ML estimation. This function has been suggested in several previous studies for GLMMs for experimental data analysis (e.g., Bolker et al., 2009; Lee and Grimm, 2018). The ML estimation for binary dependent variables requires an approximation method because there is no closed-form solution that calculates the marginal likelihood (McCulloch, 1994, 1997). The *glmer* function relies on the ML estimation, implementing Laplace approximation as the default setting.

Researchers in experimental psychology are mainly interested in hypothesis testing for their experimental condition effect, which is expressed as the fixed effect in GLMMs. As mentioned above, the Wald and LR tests are commonly used for hypothesis testing. The Wald test is commonly used for hypothesis testing of the fixed effect because it is convenient to obtain the result based on the model being evaluated (e.g., Baayen et al., 2008). The following null and alternative hypotheses regarding the experimental condition *β*_1_ can be tested using the Wald test:


(6) $$H_{0}:\beta_{1}=0\text{versus}H_{1}:\beta_{1}\neq 0$$

The test statistic is $T_{Wald}=\frac{\hat{\beta_{1}}}{SE\left(\beta_{1}\right)}$, where $\hat{\beta_{1}}$ is the estimate and *SE*(*β*_1_) is the standard error of the estimate. The test statistic is assumed to follow a standard normal distribution asymptotically. Thus, the experimental condition effect is determined to be significant if the test statistic is greater than 1.96.

The LR test compares the change in deviance (i.e., −2 times the maximum log-likelihood) between the null and alternative models. Here, the null model has the same random effect structure as the alternative model but does not include *β*_1_*x*_*k*_ term. The test statistic is expressed as follows:


(7) $$T_{LR}=-2\left[l\left(\hat{\theta_{0}}\right)-l\left(\hat{\theta_{1}}\right)\right],$$

where *θ*_0_ is the parameter set of the null model, *θ*_1_ is the parameter set of the alternative model, $l\left(\hat{\theta_{0}}\right)$ is the maximum log-likelihood of the null model, and $l\left(\hat{\theta_{1}}\right)$ is the maximum log-likelihood of the alternative model. In general, the reference distribution for the LR test is a chi-square distribution with the degree of freedom of the difference in the number of parameters. Since there are two levels in an experimental condition in the design of the current study, *χ*^2^(1) is used as a reference distribution and the test statistic is compared to it, the experimental condition effect is determined to be significant if the test statistic is greater than 3.84.

## Illustrative example

### Background

The implicit association test (IAT) was developed to measure automatic cognition toward a target category (Nosek and Banaji, 2001). In Experiment 2A of Nosek and Banaji’s (2001) study, 26 participants observed a target word selected from either the bug or fruit categories on a screen. The target word was presented simultaneously with one of the adjectives “good” or “bad.” The participants were asked to determine whether the target word matched the valence of the adjective. For illustrative purposes, we used a subset of the original dataset. Only trials where the target word was from either the bug or fruit categories (i.e., the distractor items, such as table, potato, and car were excluded) and the adjective was “good” were included in the dataset. The mismatch response to the target words from the bug category and the match response to the target words from the fruit category were coded as correct responses, and the opposite responses were coded as incorrect responses. As a result, the responses from 20 trials were included at each level in the analysis per participant. Therefore, our final dataset consisted of 1,040 data points (20 trials × 2 levels × 26 participants).

The data structure of the study is presented in Table 3. The stimuli category was used as an independent variable, and the accuracy was compared between the two levels of the category condition. All participants were exposed to both categories. There were 24 target items at each category level. Twenty items were randomly presented to each participant in each category without replacement.

**TABLE 3:** Structure of the presented stimuli (A), responses (B), and item lists (C) of Nosek and Banaji (2001).

(A)										
**Bug level**						**Fruit level**				
	**Trial 1**	**Trial 2**	**Trial 3**	**…**	**Trial 20**	**Trial 21**	**Trial 22**	**Trial 23**	**…**	**Trial 40**
1	Bug 14	Bug 13	Bug 20	…	Bug 7	Fruit 10	Fruit 15	Fruit 19	…	Fruit 1
2	Bug 23	Bug 16	Bug 10	…	Bug 19	Fruit 1	Fruit 2	Fruit 13	…	Fruit 14
⋮			⋮			⋮			⋮	
26	Bug 19	Bug 6	Bug 13	…	Bug 17	Fruit 6	Fruit 16	Fruit 13	…	Fruit 3

**(B)**										

**Bug level**						**Fruit level**				
***j***	**Trial 1**	**Trial 2**	**Trial 3**	**…**	**Trial 20**	**Trial 21**	**Trial 22**	**Trial 23**	**…**	**Trial 40**

1	NM	NM	NM	…	NM	M	NM	M	…	M
2	NM	NM	NM	…	NM	M	M	M	…	M
⋮			⋮			⋮			⋮	
26	NM	NM	NM	…	NM	M	M	M	…	M

**(C)**										

**Category**		**Item ID**								**Item**

Bug		Bug 1								Aphid
Bug		Bug 2								Ants
⋮		⋮								⋮
Bug		Bug 24								Wasp
Fruit		Fruit 1								Apple
Fruit		Fruit 2								Apricot
⋮		⋮								⋮
Fruit		Fruit 24								Watermelon

j, participant ID.

M, match; NM, non-match.

### Descriptive statistics

**FIGURE 1:**
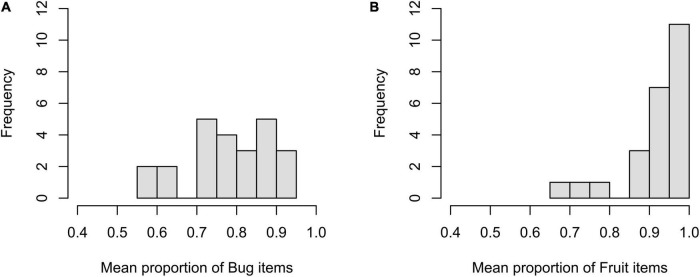
Histogram of mean proportion of the bug **(A)** and fruit **(B)** items for by-item analysis.

The mean responses were computed across participants within a level per item to illustrate item effects. A total of 48 responses were obtained from the 26 participants. The mean proportion was 0.778 (*SD* = 0.105) at the bug level and 0.923 (*SD* = 0.089) at the fruit level. As shown in Figure 1, the items at the bug level were distributed over a wide range in terms of accuracy (from 0.563 to 0.917). On the other hand, of the 24 items at the fruit level, 18 items showed an accuracy of over 0.90, ranging from 0.667 to 1.

### Generalized linear mixed-effects models results

Table 4 shows the estimates for M1 and M2, which differ noticeably in terms of the variance of the item random effect. The variance of the common item random effect in M1 was 0.219, while the variance of the level-specific item effect in M2 was 0.047 and 0.745 at the bug and fruit levels, respectively. The variance of the participant random effects and the estimate of the fixed effects were slightly larger in M2 than in M1. Furthermore, the standard error of the fixed effect was also larger in M2 than in M1.

**TABLE 4:** Estimates of the fixed and random effects of the GLMMs.

	M1		M2	
	Estimate	SE	Estimate	SE
*Fixed effect*				
Intercept [*β*_0_]	1.405	0.197	1.368	0.175
Slope [*β*_1_]	1.443	0.306	1.671	0.365
*Random effect*				
*Participant*				
Var (intercept) [$\tau_{0}^{2}$ ]	0.219		0.361	
Var (slope) [$\tau_{1}^{2}$ ]	0.370		0.254	
Corr (*s*_0*j*_, *s*_1*j*_)	−0.15		−0.11	
*Item*				
Var (item) [*ω*^2^]	0.219		NA	
Var (item1) [$\omega_{1}^{2}$ ]	NA		0.047	
Var (item2) [$\omega_{2}^{2}$ ]	NA		0.745	

M1, Common item random effect model; M2, Level-specific item random effect model; NA, not applicable.

The Wald test showed the experimental condition effect to be statistically significant in both models, *Z*s = 4.723 and 4.572 for M1 and M2, respectively; *p*s < 0.001. An LR test also showed convergent results with the Wald test, *χ*^2^(1)s = 21.045 and 23.590 for M1 and M2, respectively; *p*s < 0.001.

### Discussion

The case outlined above shows an example of an NRI design. The items could not be repeated between the levels because of the nature of the stimuli. Both descriptive statistics and GLMM estimates showed the heterogeneity of the item effects between the levels of the experimental conditions. However, the common item random effect model did not model such heterogeneity, and the estimate for the variance of the item random effect had a middle value of the variances between the two levels. Nevertheless, the consequence of the misspecification of the random effects structure on hypothesis testing of fixed effects was not critical because the effect size was considerable. In the following simulation study, we captured the number of participants, the number of items, and the random effect structure from the example above. However, we reduced the magnitude of the fixed effect.

## Simulation study

A simulation study was designed to investigate the inferential qualities (Type I error rate and power) of the two GLMMs in an NRI design. The R script used in this simulation study was uploaded to the OSF repository (see section “Data availability statement”). Thus the entire study is repeatable.

### Study design

The common item random effect model (M1) and level-specific item random effect model (M2) were used to generate data. For fixed conditions, the magnitude of *β*_0_ was set to 1, indicating that the mean response was 0.731 on the proportion scale at the first level of the experimental condition. The parameters for the person random effects were $\tau_{0}^{2}$ = 0.40, $\tau_{1}^{2}$ = 0.25, and Corr (*s*_0*j*_, *s*_1*j*_) = −0.30.

Four varying conditions were fully crossed, yielding 48 (=2 × 2 × 4 × 3) conditions. The number of participants (*J*) was selected as 25 and 50, and the number of items at each level (*I*) was 12 and 24. The numbers of participants and items were chosen from a simulation study in Barr et al. (2013). Four magnitude levels of *β*_1_ were chosen: 0, 0.2, 0.5, and 0.8. The value of 0 meant there was no effect, and the values of 0.2, 0.5, and 0.8 reflected small, medium, and large effect sizes, respectively.^1^ These parameters indicate that the mean proportions were 0.731, 0.769, 0.818, and 0.858 at the second level of the experimental condition. The variance of the item random effect had three levels: homogenous, [$\omega_{1}^{2},\omega_{2}^{2}$ ] = [0.2, 0.2], and two heterogeneous levels, [$\omega_{1}^{2},\omega_{2}^{2}$ ] = [0.2, 0.4], [0.05, 0.75]. That is, the data generating model was M1 at the homogenous level and M2 at the heterogeneous level.

Five thousand replications were simulated for each condition. We performed a total of six tests for each replication and compared the results. Hypothesis testing was performed by Wald and LR tests were performed on *β*_1_ in M1 and M2, respectively. Additionally, two *F*-tests were performed using the ANOVA framework. We reported the results of the by-participant (*F*1) and *F*1/*F*2 tests. The nominal significance level for all tests was 0.05.

### Evaluation measures

We evaluated the Type I error rate and power. The Type I error rate was defined as the proportion of the fixed effect incorrectly identified by the Wald and LR tests in the *β*_1_ = 0 condition. Power was defined as the proportion of the fixed effect correctly identified by the hypothesis testing method in the *β*_1_ = 0.2, 0.5, and 0.8 conditions. As indicators of a good statistical test, the values for the Type I error rate should be close to α = 0.05 and the values for power should be close to 1. As a rule of thumb, a Type I error rate of 0.08 or lower and power of 0.80 or higher are considered satisfactory.

### Results

In Table 5, the Type I error rate and power according to the simulation conditions are reported. The results are reported separately for M1 and M2 as the true data-generating model.

**TABLE 5:** Type I error rate (*β*_1_ = 0) and power (*β*_1_ > 0).

				ANOVA		M1		M2	
*β*_1_	*J*	*I*	[$\omega_{1}^{2},\omega_{2}^{2}$ ]	F1	F1/F2	Wald	LR	Wald	LR
0	25	12	[0.2, 0.2]	0.130	0.059	0.067	0.060	0.063	0.056
0	25	12	[0.2, 0.4]	0.173	0.063	0.070	0.064	0.066	0.060
0	25	12	[0.05, 0.75]	0.213	0.067	0.078	0.070	0.064	0.058
0	25	24	[0.2, 0.2]	0.110	0.069	0.061	0.055	0.061	0.055
0	25	24	[0.2, 0.4]	0.161	0.079	0.068	0.061	0.063	0.057
0	25	24	[0.05, 0.75]	0.206	0.087	0.070	0.062	0.054	0.048
0	50	12	[0.2, 0.2]	0.201	0.054	0.067	0.059	0.063	0.057
0	50	12	[0.2, 0.4]	0.264	0.057	0.071	0.062	0.068	0.061
0	50	12	[0.05, 0.75]	0.323	0.067	0.085	0.074	0.071	0.060
0	50	24	[0.2, 0.2]	0.187	0.077	0.064	0.059	0.065	0.059
0	50	24	[0.2, 0.4]	0.229	0.068	0.062	0.057	0.059	0.054
0	50	24	[0.05, 0.75]	0.323	0.092	0.072	0.067	0.063	0.057
0.2	25	12	[0.2, 0.2]	0.202	0.099	0.118	0.107	0.109	0.099
0.2	25	12	[0.2, 0.4]	0.210	0.090	0.107	0.096	0.111	0.100
0.2	25	12	[0.05, 0.75]	0.202	0.071	0.084	0.077	0.096	0.087
0.2	25	24	[0.2, 0.2]	0.243	0.180	0.169	0.161	0.168	0.160
0.2	25	24	[0.2, 0.4]	0.225	0.140	0.135	0.127	0.150	0.144
0.2	25	24	[0.05, 0.75]	0.187	0.093	0.095	0.088	0.137	0.134
0.2	50	12	[0.2, 0.2]	0.327	0.135	0.167	0.150	0.161	0.144
0.2	50	12	[0.2, 0.4]	0.341	0.109	0.138	0.128	0.145	0.131
0.2	50	12	[0.05, 0.75]	0.318	0.080	0.109	0.100	0.126	0.115
0.2	50	24	[0.2, 0.2]	0.398	0.230	0.225	0.213	0.220	0.208
0.2	50	24	[0.2, 0.4]	0.359	0.152	0.164	0.158	0.187	0.179
0.2	50	24	[0.05, 0.75]	0.290	0.096	0.116	0.114	0.158	0.153
0.5	25	12	[0.2, 0.2]	0.532	0.383	0.411	0.387	0.393	0.366
0.5	25	12	[0.2, 0.4]	0.482	0.304	0.330	0.306	0.339	0.320
0.5	25	12	[0.05, 0.75]	0.392	0.232	0.259	0.245	0.300	0.291
0.5	25	24	[0.2, 0.2]	0.724	0.656	0.635	0.616	0.621	0.604
0.5	25	24	[0.2, 0.4]	0.644	0.531	0.518	0.503	0.556	0.543
0.5	25	24	[0.05, 0.75]	0.494	0.358	0.367	0.360	0.489	0.484
0.5	50	12	[0.2, 0.2]	0.752	0.519	0.556	0.529	0.547	0.519
0.5	50	12	[0.2, 0.4]	0.687	0.384	0.438	0.415	0.460	0.435
0.5	50	12	[0.05, 0.75]	0.566	0.266	0.333	0.317	0.387	0.367
0.5	50	24	[0.2, 0.2]	0.911	0.808	0.799	0.786	0.794	0.778
0.5	50	24	[0.2, 0.4]	0.843	0.651	0.670	0.660	0.700	0.689
0.5	50	24	[0.05, 0.75]	0.691	0.436	0.515	0.508	0.618	0.604
0.8	25	12	[0.2, 0.2]	0.847	0.736	0.757	0.730	0.734	0.705
0.8	25	12	[0.2, 0.4]	0.787	0.629	0.650	0.628	0.659	0.641
0.8	25	12	[0.05, 0.75]	0.696	0.518	0.544	0.526	0.612	0.605
0.8	25	24	[0.2, 0.2]	0.969	0.954	0.944	0.937	0.939	0.931
0.8	25	24	[0.2, 0.4]	0.934	0.890	0.882	0.871	0.894	0.885
0.8	25	24	[0.05, 0.75]	0.855	0.760	0.772	0.763	0.858	0.858
0.8	50	12	[0.2, 0.2]	0.971	0.879	0.899	0.888	0.896	0.880
0.8	50	12	[0.2, 0.4]	0.935	0.759	0.803	0.783	0.817	0.799
0.8	50	12	[0.05, 0.75]	0.861	0.615	0.689	0.677	0.745	0.728
0.8	50	24	[0.2, 0.2]	0.998	0.993	0.991	0.990	0.991	0.988
0.8	50	24	[0.2, 0.4]	0.994	0.956	0.963	0.959	0.969	0.967
0.8	50	24	[0.05, 0.75]	0.963	0.858	0.909	0.906	0.948	0.941

M1, common item random effect model; M2, level-specific item random effect model; *β*_1_, magnitude of experimental condition; J, number of participants; I, number of items; [$\omega_{1}^{2},\omega_{2}^{2}$ ], variances of item random effects.

#### When the item effect is homogenous in the true model

##### Type I error rate

**FIGURE 2:**
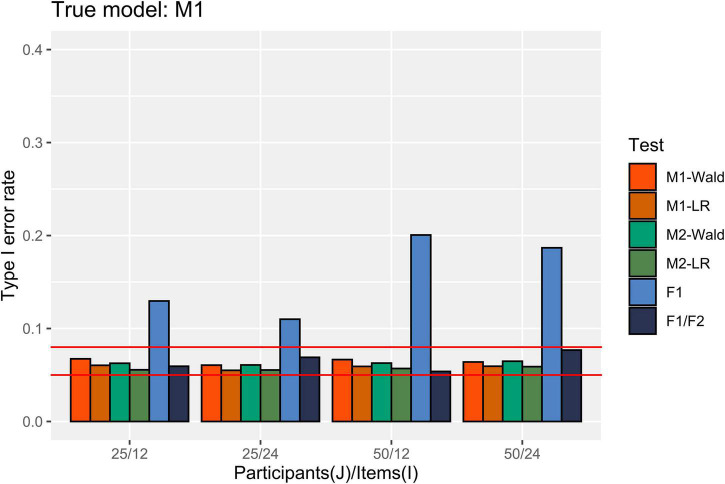
Type I error rate in the conditions where [$\omega_{1}^{2},\omega_{2}^{2}$ ] = [0.2, 0.2]. The two auxiliary lines indicate 0.05 and 0.08.

In Figure 2, the Type I error rate results are presented when M1 is the true data-generating model. M1 and M2 showed an appropriate Type I error rate at all participant (*J*) levels and numbers of items (*I*) in our simulation conditions. In addition, both models showed an error rate of less than 0.08 in the null hypothesis test using the Wald and LR tests. The performance of the *F*1/*F*2 test was comparable to that of the GLMMs. In contrast, the *F*1 test showed a high Type I error rate under all simulation conditions. The error rate always exceeded 0.1, and it increased to 0.2 when *J* = 50 and *I* = 12.

##### Power

**FIGURE 3:**
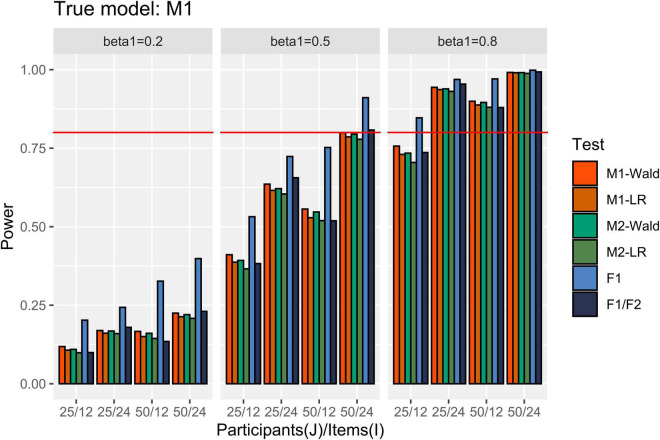
Power in the conditions where [$\omega_{1}^{2},\omega_{2}^{2}$ ] = [0.2, 0.2]. The auxiliary line indicates 0.80.

In Figure 3, the power results are shown when M1 is the true model. M1 and M2 detected more than 80% of *β*_1_ of 0.8, except for the simulation conditions where *J* = 25 and *I* = 12. When *β*_1_ = 0.5, both M1 and M2 showed power close to 0.8 only under the maximal number of participants (50) and items (24). When *β*_1_ = 0.2, the power was less than 0.222 under our simulation conditions. M1 showed higher power than M2 when the number of items and participants were small. In addition, the Wald test showed higher power than the LR test when the number of items was small within the same model. For example, under the condition that *J* = 25, *I* = 12, and *β*_1_ = 0.5, the power of Wald and LR tests in M1 and the power of Wald and LR tests in M2 were 0.411, 0.387, 0.392, and 0.366, respectively. The difference in power between the models and tests decreased as the number of participants and items increased. The *F*1/*F*2 test yielded lower power than the GLMMs when *I* = 12. The power of *F*1 was always higher than that of the other tests.

#### When the item effect is heterogenous in the true model

##### Type I error rate

**FIGURE 4:**
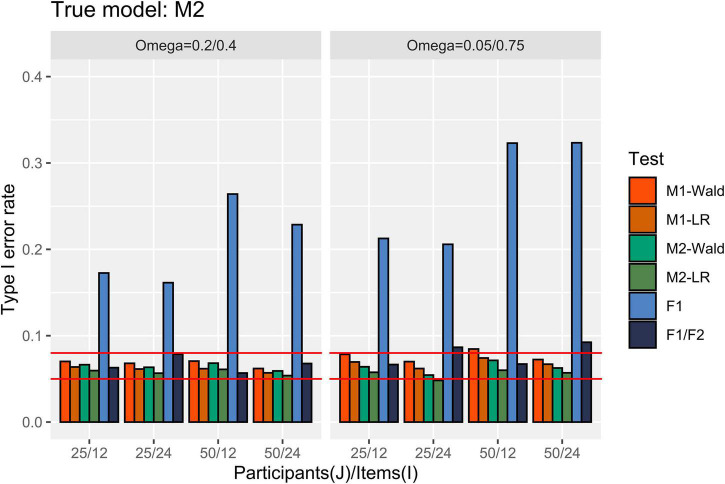
Type I error rate in the conditions where [$\omega_{1}^{2},\omega_{2}^{2}$ ] = [0.2, 0.4] and [0.05, 0.75]. The two auxiliary lines indicate 0.05 and 0.08.

Figure 4 presents the Type I error rate in the condition where M2 is the true model. M2 showed an appropriate Type I error rate for all numbers of participants and items. When [$\omega_{1}^{2},\omega_{2}^{2}$ ] = [0.05, 0.75] the Type I error rate of M1 exceeded 0.08 only when the Wald test was used under the condition with *J* = 50, *I* = 12. The *F*1/*F*2 test showed an error rate of over 0.08 under the condition where *I* = 24 and [$\omega_{1}^{2},\omega_{2}^{2}$ ] = [0.05, 0.75]. The *F*1 test showed an error rate exceeding 0.15 in all conditions.

##### Power

**FIGURE 5:**
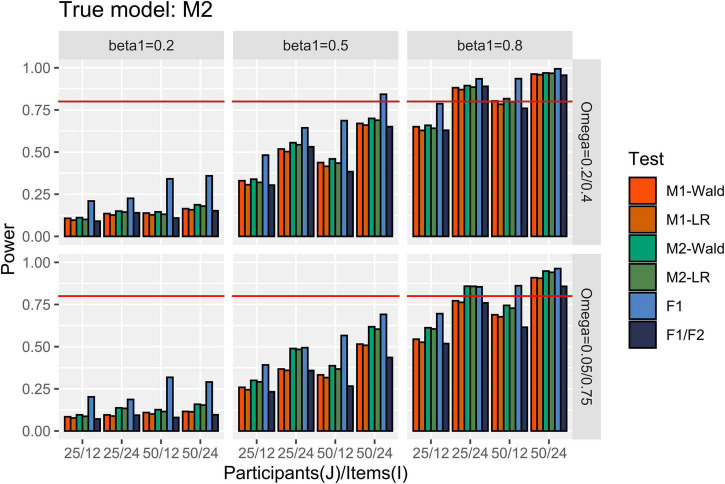
Power in the conditions where [$\omega_{1}^{2},\omega_{2}^{2}$ ] = [0.2, 0.4] and [0.05, 0.75]. The auxiliary line indicates 0.80.

Figure 5 shows the power results for the conditions in which M2 is the true model. When [$\omega_{1}^{2},\omega_{2}^{2}$ ] = [0.2, 0.4], the GLMMs successfully detected more than 80% of *β*_1_ of 0.8 when *I* = 24. At *I* = 12, only exceeded 80% when *J* = 50 and using the Wald test. When [$\omega_{1}^{2},\omega_{2}^{2}$ ] = [0.05, 0.75] and *I* = 24, M2 showed higher than 80% power to detect the *β*_1_ of 0.8, regardless of the number of participants. M1 failed to reach 80% power to detect the same magnitude of *β*_1_ when *J* = 25 and *I* = 24. The difference in power between GLMMs with common item effect and level-specific item effect was larger when *I* = 24 than when *I* = 12, and this difference is more pronounced with a larger heterogeneity in item effect. For example, under the condition that *J* = 25, *I* = 24, and *β*_1_ = 0.5, the power difference was 0.256 while the difference in power was 0.041 under the condition where *J* = 25, *I* = 12, and *β*_1_ = 0.5. When [$\omega_{1}^{2},\omega_{2}^{2}$ ] = [0.2, 0.4], the performance of the *F*1/*F*2 test was comparable to that of the GLMMs. Under the conditions where [$\omega_{1}^{2},\omega_{2}^{2}$ ] = [0.05, 0.75], the *F*1/*F*2 test performance was comparable to M1 when *J* = 25 but showed lower power than M1 when *J* = 50. Finally, *F*1 showed higher power than the other tests.

### Discussion

The following conclusions were drawn based on the simulation results. First, when the item effects are heterogeneous in NRI design data, fitting a model with the common item random effect causes a reduction in power, which is more pronounced when the number of items is large. However, no evidence of an increase in the Type I error rate was found in our simulation conditions.

Second, if the variance of the item effects of the two levels of an experimental condition is equivalent, the decrease in power due to over-specification is not that severe. Two approaches have been proposed for model selection in linear mixed-effects models (LMMs) for analyzing experimental data. Barr et al. (2013) suggested using the most complex model possible in the study design. The rationale behind the “maximal model” approach is that hypothesis testing from an experimental design can be seen as a kind of confirmatory data analysis. Using a maximal model corresponds to the practice of model specification in the analysis of variance framework. In contrast, Matuschek et al. (2017) suggested that experimental researchers select a model to analyze their data through comparing models in terms of the structure of the random effects. In the worst-case scenario of their simulation study, the power of the maximal model (i.e., a model with random slopes for both participants and items) was lower than that of the random intercept-only model by 0.089. This result is inconsistent with our simulation result, where the maximum difference in power at the homogenous level between M1 and M2 was 0.034 (LR test; *J* = 25, *I* = 12, and *β*_1_ = 0.8). However, the current study’s GLMM-based simulation results cannot be directly compared with Matuschek et al. (2017), who studied LMMs.

Third, we found that Wald and LR tests performed equally for hypothesis testing in GLMMs for experimental data analysis. The differences in Type I error rate and power were not significant between the tests in our simulation conditions, and the effect of the random effect’s misspecification on these measures was also similar. The Wald test showed a higher power than the LR test in the small sample size. However, this difference should be interpreted with caution because our experimental conditions were limited.

Lastly, the most problematic point when using ANOVA in NRI designs is the highly inflated Type I error rates. Regardless of the homogeneity of the item effects, the by-participant analysis revealed unsatisfactory Type I error rates. On the other hand, *F*1/*F*2 analysis showed lower power than M1 when the variance of the item random effect between levels was not equivalent.

## General discussion

In the current study, we drew attention to a type of experimental design (NRI) frequently used by researchers in experimental psychology and proposed a model that considers the characteristics of the data obtained from this type of design. The necessity of this model was demonstrated through an empirical dataset, and the performance of hypothesis testing was compared between the proposed and existing models via a simulation study. The implications and limitations of this study are as follows.

### Implications

The current study showed that NRI designs are not uncommon using a literature survey and an illustrative dataset example. The NRI design is an experimental design that can be considered when there is no overlap of features between the levels of experimental conditions. It can be used in various subfields such as perception (e.g., category, color, and the number of objects), social cognition (e.g., vignettes or scenarios from different contexts), and learning and memory (e.g., items selected from item pools) in experimental psychology.

This study pointed out the shortcomings of the existing methods for analyzing NRI design data and showed that the newly proposed model could overcome this shortcoming. For example, existing methods cannot capture the heterogeneity of the dependent variable originating from the item effect. On the other hand, the level-specific item random effects model considers all effects that may affect the dependent variable.

The simulation study confirmed that the proposed model performed better than other methods, given the heterogeneity of item effects. In addition, this paper provides information on the number of items and the participants for successful effect detection. For example, in the NRI design, if the size of the item effect differs between levels, 25 participants and 24 items are required to detect a large effect size successfully. On the other hand, we suggest that sample sizes be larger than those used in our study to detect a medium effect size successfully.

### Limitations and future directions

This study proposes a new model specification that overcomes the limitations of the current GLMMs due to the NRI design. In line with the illustrative example dataset and previous simulation studies (Baayen et al., 2008; Barr et al., 2013; Luke, 2017; Matuschek et al., 2017), we limited the experimental design to a within-participant design with a single factor of two levels. However, the design in the current study is too simple to apply to psychological research in practice, although the proposed model is logically applicable for complex experimental designs. The performance of GLMMs in more complex experimental designs should be examined in future studies. One issue in applying GLMMs in complex experimental designs is the problem of establishing a random effect structure. The number of parameters of GLMMs with full participant random effect structures increases rapidly as the number of responses from one participant increases. For example, the number of parameters is 18 (4 for *β* s, 10 for *τ* s, and 4 for ω s) when M2 is applied to datasets from a 2-by-2 within-participant design. In contrast, the total number of parameters was 7 (2 for *β* s, 3 for *τ* s, and 2 for *ω* s) in the current study context. The more complex the model, the larger the sample size required for a reliable estimation. To cope with model complexity, experimental researchers may refer to options for covariance structures designed for longitudinal studies [e.g., compound symmetry (CS) and first-order autoregressive (AR1); Littell et al., 2000]. Therefore, it is necessary for experimental researchers to study how to specify the model, select the random effect structure, and set the appropriate number of participants and items in various experimental designs.

All simulation studies make assumptions regarding true models. A normal or multivariate normal distribution was assumed for the random effects in this study. In GLMMs, the fixed effect estimate is biased when the random effect distribution is incorrectly specified (Verbeke and Lesaffre, 1997; Litière et al., 2007). Further research is required to investigate the robustness of GLMMs for NRI designs in the case of non-normality for random effects.

Finally, studies on alternative estimation and hypothesis testing methods are needed. Only ML estimation and the null-hypothesis significance testing (NHST) approach were dealt with in this study. However, in psychology, interest in Bayesian modeling is increasing, as shown in the 2018 special issue of the *Psychonomic Bulletin and Review* (Vandekerckhove et al., 2018). GLMMs can be well estimated using the Bayesian approach, in which a random effect is regarded as a type of prior distribution (Gelman and Hill, 2007). In addition, an increasing number of statistical software packages include Bayesian estimation as an option [e.g., the MCMCglmm package in R (Hadfield, 2010) and Mplus (Muthén and Muthén, 2017)]. In a study, several software packages for GLMMs were fitted to one experimental dataset, and the results were compared (Lee and Grimm, 2018). The software packages relying on ML and Bayesian estimations produced comparable results regarding the estimates of the fixed and random effects and their standard errors. However, for NRI designs, performance evaluation of GLMMs according to the estimation method has not yet been investigated. Therefore, GLMMs for NRI designs also need to be discussed from a Bayesian perspective.

## Data availability statement

The dataset and R script used for the illustrative example and simulation study sections can be accessed *via* the OSF repository of the corresponding author (https://osf.io/kryqu/).

## Author contributions

SH designed a simulation study, performed the simulation and statistical analysis, and wrote the manuscript. WL initiated the project, developed the model, and wrote the manuscript. Both authors contributed to the manuscript revision, read, and approved the submitted version.

## Footnotes

1 The effect size is not consistent with the guideline of Cohen (1988). The logit difference is transformed to Cohen’s d using $d=logit\times \frac{\sqrt{3}}{\pi }$ . The effect sizes manipulated in our experiment correspond to [0, 0.110, 0.276, 0.441] in the unit of Cohen’s d.
